# Risk Factors and Outcomes of Disseminated Nocardiosis Across Host Risk Groups

**DOI:** 10.1093/ofid/ofag008

**Published:** 2026-01-12

**Authors:** Maria Vega Brizneda, Cyndee Miranda, Eric Cober, Anisha Misra, Susan Harrington, Zachary A Yetmar

**Affiliations:** Department of Infectious Disease, Cleveland Clinic, Cleveland, Ohio, USA; Department of Infectious Disease, Cleveland Clinic, Cleveland, Ohio, USA; Department of Infectious Disease, Cleveland Clinic, Cleveland, Ohio, USA; Department of Pathology and Laboratory Medicine, Cleveland Clinic, Cleveland, Ohio, USA; Department of Pathology and Laboratory Medicine, Cleveland Clinic, Cleveland, Ohio, USA; Department of Infectious Disease, Cleveland Clinic, Cleveland, Ohio, USA

**Keywords:** brain abscess, dissemination, immunocompromised, mortality, *nocardia*

## Abstract

**Background:**

Nocardiosis primarily affects immunocompromised hosts and those with chronic pulmonary disease but can also occur in immunocompetent patients. Predictors of dissemination and its role in long-term mortality remain unclear.

**Methods:**

We conducted a retrospective cohort study of adults with nocardiosis diagnosed from January 1, 2010 to December 31, 2023. Patients were categorized into 3 groups: immunocompromised, immunocompetent with chronic lung disease, and immunocompetent without chronic lung disease. We evaluated risk factors associated with dissemination at the time of diagnosis and predictors of 1-year mortality. Multivariable logistic regression identified risk factors for dissemination. Cox regression assessed predictors of 1-year mortality.

**Results:**

Among 232 patients, 44 (19.0%) had disseminated infection and 36 (15.5%) died within 1-year. Dissemination was more common among patients who were immunocompromised (odds ratio ([OR] 6.26, 95% confidence interval [CI] 2.26–20.53) or immunocompetent without chronic lung disease (OR 5.09, 95% CI 1.75–17.15). Lymphopenia and infection with *Nocardia farcinica* were also independently associated with dissemination. Dissemination was not associated with mortality overall (hazard ratio [HR] 1.58, *P* = .222), though interaction analysis revealed that dissemination was significantly associated with 1-year mortality only in immunocompetent patients with chronic lung disease (HR 9.43, 95% CI 1.73–51.52).

**Conclusions:**

Immunocompromised patients and those without chronic lung disease are at increased risk for disseminated nocardiosis. While dissemination alone is not predictive of 1-year mortality overall, it is directly associated with mortality among immunocompetent patients with chronic lung disease. These findings highlight the need for tailored prognostic assessment and management in this subgroup.

## Teaser

Nocardiosis affects immunocompromised hosts, individuals with chronic pulmonary disease, and apparently immunocompetent patients. Risk of dissemination and its impact on mortality varies between risk groups categories. Our findings highlight the need for tailored prognostic assessment and management by subgroup.

*Nocardia* species are partially acid-fast, filamentous, aerobic gram-positive bacteria ubiquitous in the environment [1]. Nocardiosis primarily affects immunocompromised individuals and those with chronic pulmonary disease, but also occur in immunocompetent hosts [2]. The primary form of nocardiosis is predominantly pulmonary due to inhalation. However, its presentations range widely from cutaneous nocardiosis by direct inoculation to the skin to severe dissemination of multiple sites including the central nervous system (CNS), which can result in significant morbidity and mortality.

Disseminated nocardiosis is traditionally associated with worse outcomes and increased treatment complexity [3]. Despite dissemination's clinical importance, its risk factors and outcomes are incompletely characterized. Species such as *Nocardia farcinica* have been associated with higher rates of dissemination, but clinical predictors remain understudied [4]. Moreover, it is unclear if disseminated infection is associated with mortality, as some studies describe an association while others find no independent effect when considering comorbidities and immune status [4–8]. Whether dissemination itself or other confounding factors drives these associations remains unknown. Prior multivariable analyses have been limited to immunocompromised populations [4, 9, 10] and have not comprehensively evaluated all distinct risk groups.

Our objective is to identify risk factors associated with dissemination, specifically to help determine who may benefit from brain imaging. Additionally, we seek to evaluate predictors of 1-year mortality, including the prognostic impact of dissemination based on *Nocardia* risk groups.

## METHODS

### Study Design

We performed a retrospective cohort study of adults with nocardiosis diagnosed from January 1, 2010 to December 31, 2023. Patients with microbiologic testing showing *Nocardia* species or *Nocardia*-associated diagnosis ICD-10 codes (A43.X) were identified and manually screened through predetermined criteria. Inclusion criteria were age ≥18 years at the time of diagnosis and nocardiosis, defined as both the identification of Nocardia species by culture, Nocardia-specific polymerase chain reaction, or 16S ribosomal RNA (rRNA) gene sequencing, and presence of clinical signs, symptoms, and/or radiologic findings consistent with *Nocardia* infection. Exclusion criteria were *Nocardia* colonization (microbiologic testing with *Nocardia* species but without signs, symptoms, or radiologic findings to suggest clinical infection) and no microbiologic confirmation of *Nocardia*.

Data on included patients were manually extracted from electronic medical records. These included demographics, comorbid conditions, clinical and imaging characteristics at presentation, treatment variables, and outcomes. Data were collected and managed using REDCap electronic data capture tools hosted at Cleveland Clinic [11, 12].

### Identification and Susceptibility Testing

*Nocardia* species were isolated from routine bacteriology, fungal, and mycobacterial cultures. Additionally, a dedicated *Nocardia* culture utilizing buffered charcoal yeast extract agar was available through June of 2023. Clinicians were thereafter directed to mycobacterial culture for recovery of *Nocardia* species. After discontinuation of the dedicated Nocardia medium, clinicians were advised to submit specimens for mycobacterial culture, which has comparable performance for recovering Nocardia species and was incorporated into routine laboratory workflows. Mycobacterial culture included BD BACTEC Mycobacterial Growth Indicator Tube (MGIT) incubated in the MGIT 960 Automated System (Becton, Dickinson and Company, Franklin Lakes, NJ) and Middlebrook 7H11 and 7H11 Selective agar incubated at 35°–37°C for up to 6 weeks. *Nocardia* species were identified by sequence analysis of the 16S rRNA gene using either pyrosequencing of a 35–43 bp hypervariable region [13] or Sanger sequencing of approximately the first 500-bp. Antimicrobial susceptibility testing (AST) was performed by a referral laboratory by broth microdilution and interpreted according to the Clinical and Laboratory Standards Institute guidelines [14, 15]. AST was performed on request.

### Definitions

The date of *Nocardia* diagnosis was the date of first culture or molecular test acquisition with *Nocardia*. Disseminated infection was defined as isolated CNS involvement or involvement of at least 2 noncontiguous organs as defined in prior studies [3–5]. Secondary sites of infection could be inferred by imaging compatible with nocardiosis if a primary site had microbiologic confirmation (eg, brain imaging suggestive of CNS involvement with confirmed pulmonary nocardiosis). Immunocompromised status was defined as receipt of ≥20 mg/day of prednisone-equivalent corticosteroids, receipt of other immunosuppressive medications, human immunodeficiency virus (HIV) infection with CD4 count <200 cells/µL, and/or being within 100 days of hematopoietic stem cell transplantation (HSCT) before *Nocardia* diagnosis consistent with prior nocardiosis cohorts [6, 7]. Immunosuppressant use was assessed 28 days prior to *Nocardia* diagnosis. Corticosteroid dosing was the most recent recorded dose in the past 28 days, converted to prednisone-equivalent dose. Trimethoprim–sulfamethoxazole (TMP–SMX) prophylaxis was assessed on the date of initial presentation. Active malignancy included radiographic or laboratory evidence of malignancy or the receipt of antineoplastic chemotherapy within 28 days of diagnosis. Patients with chronic lung disease receiving <20 mg/day prednisone or with remote malignancy or HSCT were categorized within the chronic-lung-disease group to reflect their predominant pulmonary phenotype. Laboratory values were assessed on the date of diagnosis or the most recent prior measurement. CNS symptoms included headache, confusion, paresthesia, focal weakness, and seizure-like activity. Chronic lung disease was comprised of chronic obstructive pulmonary disease (COPD), interstitial lung disease, bronchiectasis, and other pulmonary conditions requiring ongoing pulmonology follow-up aligned with previously published risk-group definitions [8].

### Statistical Analysis

Baseline characteristics were described with summary statistics, including median (interquartile range [IQR]) for continuous variables and number (percentage) for categorical variables. Patients were grouped into 3 primary risk categories: immunocompromised, chronic lung disease without immunocompromise, and those without chronic lung disease or immunocompromise. The primary outcome was disseminated infection, assessed at the time of initial nocardiosis diagnosis. This was analyzed by multivariable logistic regression. The secondary outcome was 1-year mortality, starting from the date of the nocardiosis diagnosis. Surviving patients were censored at 1-year or the date of last follow-up, whichever occurred first. Survival was illustrated with Kaplan–Meier curves, compared by log-rank test. One-year mortality was assessed by multivariable Cox regression. Variables were chosen for inclusion in the regression models a priori based on prior literature, clinical significance, and hypothesized associations. Risk groups were coded using dummy variables (immunocompromised; immunocompetent without chronic lung disease), with chronic-lung-disease as the reference category. To assess a differential effect of dissemination on mortality between risk categories, a secondary analysis was performed with an interaction term between risk category and dissemination status. To avoid overfitting the interaction analysis, risk category and dissemination status were not adjusted for other variables. Three preplanned sensitivity analyses were performed. In a post-hoc model, *N. nova* was included as an additional species variable. Due to the possibility that patients without chronic lung disease or apparent immunocompromise had undiagnosed immunocompromising conditions [16, 17], we repeated our analyses with the risk categories regrouped as those with chronic lung disease but without immunocompromise and all other patients (ie, a combined group of immunocompromised patients and those without chronic lung disease or apparent immunocompromised status). Due to the possibility of misclassification bias, we repeated our analyses after excluding patients who did not have CNS imaging. The third sensitivity analysis excluded immunocompetent patients with isolated cutaneous nocardiosis, as these patients likely had a different portal of entry from most other patients. The proportional hazards assumption from Cox models was assessed by Schoenfeld residuals. All analyses were performed using R version 4.4.1 (R Foundation for Statistical Computing, Vienna, Austria).

## RESULTS

### Baseline Characteristics

Of the 232 patients who met inclusion criteria, the median age was 66.5 years (IQR 56.6–73.4), and 122 (52.6%) patients were female. Two patients (0.9%) were diagnosed with 16S rRNA sequencing alone without culture growth; both had compatible clinical and radiologic findings supporting true infection. Blood cultures were obtained in 154 of 232 patients (66%), with *Nocardia* bacteremia detected in 11 (7.1%). Among the 3 risk groups, 88 (37.9%) patients were immunocompromised, 82 (35.3%) were immunocompetent but had chronic lung disease, and 62 (26.7%) were immunocompetent and without chronic lung disease (Table 1). Most immunocompromised patients were receiving immunosuppressing medications (N = 86, 97.7%). Three patients were living with HIV, 2 of whom had suppressed viral loads but also suppressed CD4 cell counts (12 and 182 cells/µL, respectively) and 1 had a concurrent kidney transplant. Among 25 patients with active malignancy, 18 (72%) had solid tumors and 7 (28%) had hematologic malignancies. No patients had a primary immunodeficiency. Most patients with chronic lung disease had bronchiectasis (N = 42, 51.2%) or COPD (N = 29, 35.4%). Twenty (8.6%) patients were taking TMP–SMX prophylaxis at diagnosis. Prophylactic doses included 800–160 mg thrice-weekly (N = 11, 55.0%), 400–80 mg daily (N = 2, 10.0%), 400–80 mg thrice-weekly (N = 4, 20.0%), and 400–80 mg twice-weekly (N = 3, 15.0%).

**Table 1.** Baseline Characteristics of Patients With Nocardiosis, Grouped by Risk Category

	Chronic Lung Disease (N = 82)	Immunocompetent Without Lung Disease (N = 62)	Immunocompromised (N = 88)	Total (N = 232)
Age, years	71.1 (62.3, 75.7)	64.5 (55.5, 71.7)	64.5 (54.2, 70.0)	66.5 (56.6, 73.4)
Sex	…	…	…	…
Female	53 (64.6%)	29 (46.8%)	40 (45.5%)	122 (52.6%)
Male	29 (35.4%)	33 (53.2%)	48 (54.5%)	110 (47.4%)
Race	**…**	…	…	…
Asian	2 (2.4%)	0 (0.0%)	0 (0.0%)	2 (0.9%)
Black or African American	3 (3.7%)	3 (4.8%)	14 (15.9%)	20 (8.6%)
White	77 (93.9%)	59 (95.2%)	68 (77.3%)	204 (87.9%)
Other	0 (0.0%)	0 (0.0%)	6 (6.8%)	6 (2.6%)
Hispanic or Latino ethnicity	3 (3.7%)	6 (9.7%)	7 (8.0%)	16 (6.9%)
Charlson comorbidity index	1.0 (1.0, 2.0)	0.0 (0.0, 2.0)	2.0 (1.0, 3.0)	1.0 (1.0, 3.0)
Chronic pulmonary disease	82 (100.0%)	0 (0.0%)	34 (38.6%)	116 (50.0%)
Bronchiectasis	42 (51.2%)	0	5 (14.7%)	47 (40.5%)
COPD	29 (35.4%)	0	12 (35.3%)	41 (35.3%)
ILD	4 (4.9%)	0	16 (47.1%)	20 (17.2%)
Other	7 (8.5%)	0	1 (2.9%)	8 (6.9%)
Solid organ transplant	0 (0.0%)	0 (0.0%)	34 (38.6%)	34 (14.7%)
Hematopoietic stem cell transplant	1 (1.2%)	1 (1.6%)	7 (8.0%)	9 (3.9%)
Allogeneic	0 (0.0%)	1 (100.0%)	6 (85.7%)	7 (77.8%)
Autologous	1 (100.0%)	0 (0.0%)	1 (14.3%)	2 (22.2%)
Active malignancy	5 (6.1%)	2 (3.2%)	18 (20.5%)	25 (10.8%)
Chronic kidney disease	6 (7.3%)	4 (6.5%)	34 (38.6%)	44 (19.0%)
End stage kidney disease	0 (0.0%)	1 (1.6%)	2 (2.3%)	3 (1.3%)
HIV	0 (0.0%)	0 (0.0%)	3 (3.4%)	3 (1.3%)
Immunosuppressant use within 28 days	3 (3.7%)	2 (3.2%)	86 (97.7%)	91 (39.2%)
Tacrolimus	0 (0.0%)	0 (0.0%)	35 (39.8%)	35 (15.1%)
Cyclosporine	0 (0.0%)	0 (0.0%)	3 (3.4%)	3 (1.3%)
Mycophenolate	0 (0.0%)	0 (0.0%)	30 (34.1%)	30 (12.9%)
Azathioprine	0 (0.0%)	0 (0.0%)	2 (2.3%)	2 (0.9%)
TNF-alpha inhibitor	0 (0.0%)	0 (0.0%)	5 (5.7%)	5 (2.2%)
Leflunomide	0 (0.0%)	0 (0.0%)	2 (2.3%)	2 (0.9%)
Chemotherapy	0 (0.0%)	0 (0.0%)	10 (11.4%)	10 (4.3%)
Ruxolitinib	0 (0.0%)	0 (0.0%)	1 (1.1%)	1 (0.4%)
Other immunosuppression	0 (0.0%)	0 (0.0%)	13 (14.8%)	13 (5.6%)
Corticosteroid	3 (3.7%)	2 (3.2%)	65 (73.9%)	70 (30.2%)
Daily prednisone dose, mg (N = 70)	10.0 (6.2, 10.0)	7.5 (6.2, 8.8)	15.0 (5.0, 40.0)	12.5 (5.0, 40.0)
Primary TMP–SMX prophylaxis	0 (0.0%)	1 (1.6%)	19 (21.6%)	20 (8.6%)
DS TIW	0	1 (100.0%)	10 (52.6%)	11 (55.0%)
Other	0	0 (0.0%)	3 (15.8%)	3 (15.0%)
SS daily	0	0 (0.0%)	2 (10.5%)	2 (10.0%)
SS TIW	0	0 (0.0%)	4 (21.1%)	4 (20.0%)
CNS imaging	45 (54.9%)	32 (51.6%)	70 (79.5%)	147 (63.4%)
CT head	33 (40.2%)	22 (35.5%)	48 (54.5%)	103 (44.4%)
Brain MRI	18 (22.0%)	21 (33.9%)	44 (50.0%)	83 (35.8%)
Leukocyte count (N = 214)	7.9 (6.5, 11.4)	9.7 (8.0, 12.6)	9.5 (6.1, 13.1)	8.9 (6.6, 12.5)
Neutrophil count (N = 207)	5.4 (4.0, 8.7)	7.3 (5.7, 10.6)	7.2 (4.4, 11.4)	6.8 (4.4, 10.5)
Lymphocyte count (N = 207)	1.6 (1.2, 2.1)	1.2 (0.8, 1.8)	0.6 (0.4, 1.0)	1.0 (0.6, 1.7)
Extent of infection	…	…	…	…
CNS	4 (4.9%)	11 (17.7%)	18 (20.5%)	33 (14.2%)
Non-CNS disseminated	1 (1.2%)	3 (4.8%)	7 (8.0%)	11 (4.7%)
Nondisseminated pulmonary	74 (90.2%)	12 (19.4%)	49 (55.7%)	135 (58.2%)
Isolated cutaneous	2 (2.4%)	29 (46.8%)	7 (8.0%)	38 (16.4%)
Other nondisseminated	1 (1.2%)	7 (11.3%)	7 (8.0%)	15 (6.5%)

Data are presented as median (IQR) or n (%), as appropriate.

Abbreviations: CNS, central nervous system; COPD, chronic obstructive pulmonary disease; DS, double-strength; HIV, human immunodeficiency virus; ILD, interstitial lung disease; MRI, magnetic resonance imaging; N, number; SS, single-strength; TIW, 3 times weekly; TMP–SMX, trimethoprim–sulfamethoxazole.

### Nocardia Characteristics and Dissemination

Forty-four (19.0%) patients had disseminated nocardiosis, of which 33 (75.0%) involved the CNS (Table 2). Nine (20.5%) cases of dissemination to the CNS occurred in the absence of concurrent pulmonary nocardiosis (Table 2). Among the 188 (81.0%) patients with nondisseminated infection, 135 (71.8%) had pulmonary involvement and 38 (20.2%) cutaneous involvement. Mutually exclusive sites of infection are detailed in Supplementary Table 1.

**Table 2.** Sites of Nocardiosis

	Nondisseminated (N = 188)	Disseminated (N = 44)	Total (N = 232)
Pulmonary	135 (71.8%)	35 (79.5%)	170 (73.3%)
Pleural	10 (5.3%)	6 (13.6%)	16 (6.9%)
CNS	0 (0.0%)	33 (75.0%)	33 (14.2%)
Cutaneous	38 (20.2%)	12 (27.3%)	50 (21.6%)
Osteomyelitis	2 (1.1%)	2 (4.5%)	4 (1.7%)
Arthritis	2 (1.1%)	1 (2.3%)	3 (1.3%)
Lymphadenitis	1 (0.5%)	0 (0.0%)	1 (0.4%)
Bacteremia	2 (1.1%)	9 (20.5%)	11 (4.7%)
Other	10 (5.3%)^a^	7 (15.9%)^b^	17 (7.3%)

These sites of nocardiosis are not necessarily mutually exclusive.

^a^Other sites in the nondisseminated group include one each of bursitis, keratitis, epidural abscess, intra-abdominal abscess, nephritis, mediastinitis, pyomyositis, peritonitis, sinusitis, and submandibular abscess.

^b^Other sites in the disseminated group include one each of endophthalmitis, subretinal abscess, epidural abscess, epidural and psoas abscess, hepatic abscess, pyomyositis, and retinitis.

The most common *Nocardia* species were *N. nova* (N = 61, 26.3%), *N. farcinica* (N = 40, 17.2%), and *N. cyriacigeorgica* (N = 33, 14.2%). 15 of 40 (37.5%) *N. farcinica* isolates were isolated from patients with dissemination. Twenty-two of 24 (91.7%) of *N. brasiliensis* isolates were found in patients with nondisseminated cutaneous infection and 46 of 61 (75.4%) of *N. nova* isolates were identified in those with nondisseminated pulmonary infection (Supplementary Table 2). AST was performed on 186 (82.3%) patients' isolates (Supplementary Table 3). *N. nova* infection was associated with lower odds of dissemination (odds ratio [OR] 0.35, 95% confidence interval [CI] 0.12–0.84, *P* = .029).

Rates of disseminated infection were highest in the immunocompromised group (N = 25, 28.4%), followed by immunocompetent without lung disease (N = 14, 22.6%) and chronic lung disease (N = 5, 6.1%) groups. This included 18 (20.5%), 11 (17.7%), and 4 (4.9%) with CNS involvement in the immunocompromised, immunocompetent without lung disease, and chronic lung disease groups, respectively. Rates of nondisseminated pulmonary nocardiosis were 55.7% (N = 49) in the immunocompromised, 19.4% (N = 12) in the immunocompetent without lung disease, and 90.2% (N = 74) in the immunocompetent with chronic lung disease groups. Isolated cutaneous nocardia was more common in the immunocompetent without lung disease group (N = 29, 46.8%) than in the immunocompromised (N = 7, 8.0%) or chronic lung disease groups (N = 2, 2.4%).

Among 33 patients with CNS nocardiosis, 22 (66.7%) had CNS symptoms at the time of presentation. All 4 immunocompetent patients with chronic lung disease had CNS symptoms, while only 9 of 11 (81.8%) immunocompetent patients without chronic lung disease and 9 of 18 (50.0%) immunocompromised patients had CNS symptoms.

### Treatment and Outcomes

Disseminated nocardiosis was more likely to be treated with combination therapy and multiple antibiotics than nondisseminated cases (median number of antibiotics 2 vs 1, *P* < .001). Patients with dissemination more frequently underwent procedural interventions (56.8% vs 18.1%, *P* < .001). In disseminated cases, length of therapy was longer (median 364 vs 190 days, *P* < .001) and secondary prophylaxis was more common (41.9% vs 19.2%, *P* = .010). Active empiric therapy was initiated in 214 of 232 patients (92%), typically with TMP–SMX with or without a carbapenem or amikacin. Additional treatment characteristics are in Supplementary Table 4.

On multivariable analysis, immunocompromised patients (OR 6.26, 95% CI 2.26–20.53) an immunocompetent patient without chronic lung disease (OR 5.09, 95% CI 1.75–17.15) had higher odds of dissemination than those with chronic lung disease. Infection with *N. farcinica* was also independently associated with dissemination (OR 3.66, 95% CI 1.62–8.25). Charlson comorbidity index (CCI) and TMP–SMX primary prophylaxis were not associated with dissemination (Table 3). When recategorizing the risk groups, those who were immunocompetent with chronic lung disease had lower odds of dissemination than all other patients. Sensitivity analyses excluding patients without CNS imaging and excluding immunocompetent patients with cutaneous nocardiosis found similar results (Supplementary Tables 5–6).

**Table 3.** Multivariable Logistic Regression Model of Associations With Disseminated Infection

	Odds Ratio	95% CI	*P*-Value
Immunocompetent without lung disease^a^	5.09	1.75–17.15	.004
Immunocompromised^a^	6.26	2.26–20.53	<.001
*Nocardia farcinica*	3.66	1.62–8.25	.002
Charlson comorbidity index (per 1 point increase)	1.17	0.97–1.42	.095
Primary trimethoprim–sulfamethoxazole prophylaxis	0.59	0.15–1.94	.414

Each risk category was compared with the chronic-lung-disease reference group using dummy-variable coding; direct pairwise comparisons between the 2 nonreference groups were not modeled.

Abbreviations: CI, confidence interval.

^a^Compared with the chronic lung disease group. This analysis was repeated with the initial 3-level risk category variable recategorized as “Immunocompetent with chronic lung disease”. This group showed significantly reduced odds of dissemination (odds ratio 0.18, 95% confidence interval 0.06–0.44; *P* < .001).

### Mortality

**Figure 1.**
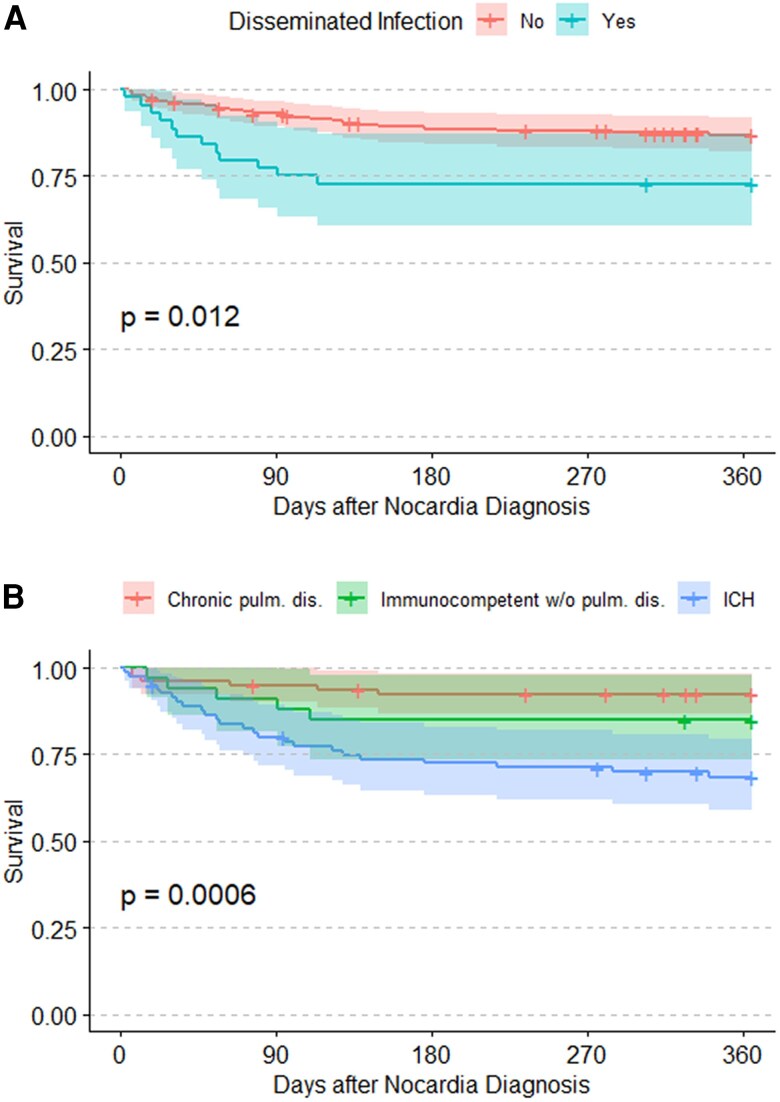
Kaplan–Meier analysis comparing survival between groups based on disseminated infection (*A*) and risk groups (*B*). The *P*-values are calculated via the log-rank test. Abbreviations: Dis., disease; Diss., dissemination; ICH, immunocompromised host; pulm., pulmonary.

Among all included patients, 36 (15.5%) patients died within 1-year of nocardiosis diagnosis. Among patients who did not die within 1 year, 22 did not have full 1-year follow-up. These 22 patients had a median post-diagnosis follow-up of 278.5 days (IQR 106.0–317.8). In Kaplan–Meier analysis, patients with disseminated infection had higher 1-year mortality than those with nondisseminated infection (27.3% vs 13.1%, *P* = .012; Figure 1*A*). Immunocompromised patients had lower 1-year survival (31.5%) compared with the immunocompetent with chronic lung disease (7.6%) and immunocompetent without chronic lung disease groups (15.2%, *P* = .0006; Figure 1*B*).

On multivariable Cox analysis, immunocompromised status (hazard ratio [HR] 3.08, 95% CI 1.22–7.76, *P* = .017) and higher CCI (HR 1.24 per point, 95% CI 1.07–1.43, *P* = .005) were associated with 1-year mortality. Dissemination (HR 1.58, 95% CI 0.76–3.32, *P* = .222), infection with *N. farcinica* (HR 1.33, 95% CI 0.58–3.03, *P* = .498), and immunocompetent status without chronic lung disease (HR 1.16, 95% CI 0.35–3.86, *P* = .808) were not predictive of mortality. When excluding patients without CNS imaging and immunocompetent patients with isolated cutaneous infection, these analyses showed similar results (Supplementary Tables 7–8).

An interaction between risk category and disseminated status was assessed in a multivariable model, which was not adjusted for other factors. The model was intentionally unadjusted to avoid overfitting and multicollinearity given the limited number of mortality events within specific subgroups. This analysis showed disseminated status was not associated with 1-year mortality among immunocompromised patients (interaction HR 1.65, 95% CI 0.73–3.73, *P* = .231) or immunocompetent patients without chronic lung disease (interaction HR 0.82, 95% CI 0.09–7.29, *P* = .855). However, dissemination was associated with 1-year mortality among immunocompetent patients with chronic lung disease (interaction HR 9.43, 95% CI 1.73–51.52, *P* = .010). One-year mortality calculated by inversed Kaplan–Meier method to account for incomplete follow-up, showed the effect of dissemination on mortality for the immunocompromised group was HR 1.65 (95% CI 0.73–3.73, *P* = .231), immunocompetent without chronic lung disease group was HR 0.82 (95% CI 0.09–7.29, *P* = .855), and immunocompetent with chronic lung disease group was HR 9.43 (95% CI 1.73–51.52, *P* = .010) **(**Table 4**)**.

**Table 4.** One-Year Mortality by Risk Category and Dissemination

Dissemination	Risk Category	One-Year Mortality	Hazard Ratio (95% CI)	*P*-Value
Yes	Immunocompromised	36.0%	8.66 (2.66–28.14)	<.001
No	Immunocompromised	26.2%	5.26 (1.76–15.73)	.003
Yes	Immunocompetent without chronic lung disease	7.1%	1.34 (0.15–11.97)	.795
No	Immunocompetent without chronic lung disease	8.4%	1.64 (0.41–6.56)	.484
Yes	Immunocompetent with chronic lung disease	40.0%	9.43 (1.73–51.52)	.010
No	Immunocompetent with chronic lung disease	5.3%	1 (reference)	–

Abbreviation: CI, confidence interval.

## DISCUSSION

In this cohort study of 232 patients with nocardiosis, we evaluated predictors of dissemination and 1-year mortality across 3 relevant risk groups. Immunocompromised patients and immunocompetent individuals without chronic lung disease had significantly higher rates of dissemination than patients solely with chronic lung disease. Chronic lung disease, particularly bronchiectasis, has been associated with increased pulmonary nocardiosis, most of whom were immunocompetent, with rare development of dissemination and low mortality [18]. Notably from our study, dissemination appeared to impact 1-year mortality differently depending on risk group. Among those with disseminated diseases, only immunocompetent patients with chronic lung disease had significantly increased mortality. Several mechanisms may explain this differential effect. Immunocompetent patients with chronic lung disease may have delays in recognition of extrapulmonary involvement, as new neurologic or systemic symptoms are often attributed to underlying pulmonary pathology. Chronic airway inflammation and impaired mucociliary clearance may further limit early pathogen eradication once dissemination occurs.

Dissemination has been previously associated with immunosuppression [4]. Our study confirmed that immunocompromised patients are at high risk for dissemination, consistent with reports in which 34%–44% of immunocompromised patients presented with dissemination [9, 10]. However, in our cohort, immunocompetent individuals without chronic lung disease had a similar risk of dissemination. This finding may be explained by unknown underlying immunodeficiencies that predispose immunocompetent individuals without chronic lung disease to dissemination [19]. Additional contributors may include subtle or undiagnosed immune defects such as anti-GM-CSF autoantibodies or other primary immune abnormalities that are increasingly recognized among patients with severe nocardiosis. Also, this subgroup often has less healthcare contact and may present later in their disease. Conversely, immunocompetent patients with chronic lung disease have lower risk of dissemination due to more frequent healthcare contact and more frequent imaging leading to early diagnosis, and/or localized immune adaptation from chronic inflammation [20].

Identifying CNS infection among at-risk individuals is important as CNS involvement has therapeutic and monitoring implications. Past studies evaluating primarily solid organ transplant or HSCT recipients found only roughly half of immunocompromised patients with CNS nocardiosis present with CNS symptoms [21, 22]. Similarly, 50% of our cohort's immunocompromised patients with CNS involvement presented with CNS symptoms. Among immunocompetent patients with or without chronic lung disease who had CNS disease in our cohort, far more presented with CNS symptoms, 100% and 81.8%, respectively. Routine brain imaging may have a low diagnostic yield among immunocompetent patients with chronic lung disease who lack neurologic symptoms; however larger studies are required to validate this observation.

Infection with *N. farcinica* was independently associated with dissemination in our cohort. This is consistent with past microbiologic and clinical studies implicating this species as a more virulent group with higher rates of dissemination or CNS disease, and mortality [6, 8, 23]. Additionally, we found other species-specific patterns, such as *N. brasiliensis* primarily causing nondisseminated cutaneous infection, and *N. nova* negatively associated with dissemination, typically presenting as localized pulmonary disease. These findings suggest *Nocardia* species identification can provide useful prognostic information in addition to species-specific antibiotic susceptibility patterns [24–27]. *N. farcinica* especially should prompt consideration of CNS involvement and aggressive empiric therapy.

Antibiotic susceptibility patterns in our cohort mirrored prior reports, with low imipenem susceptibility among *N. farcinica* isolates and reliable TMP–SMX and linezolid activity. These findings support the need for species-specific AST, particularly when empirical β-lactams are used or when CNS penetration is a concern.

Immunocompromised status and a higher burden of comorbidities were associated with higher 1-year mortality, whereas dissemination overall was not. These findings underscore the importance of patient characteristics when evaluating nocardiosis. In a multicenter cohort of 374 patients with nocardiosis, the immunocompromised population had the highest rate of mortality, which appeared to be driven by comorbidity burden and specific sites of infection [8].

Conversely, dissemination has also been inconsistently linked to mortality [5–8, 21]. Studies solely analyzing immunocompromised populations have specifically failed to show an association between dissemination and mortality [5, 7, 21]. This raises the possibility of either a differential effect between patient populations or confounding within studies enrolling both immunocompromised and immunocompetent patients. Our interaction analysis revealed a novel insight regarding how dissemination impacts mortality differently among the groups. In immunocompetent patients with chronic lung disease, dissemination was associated with significantly increased 1-year mortality. In contrast, dissemination did not significantly increase mortality in immunocompromised patients or immunocompetent individuals without lung disease. There are several potential explanations for this differential effect. The association between dissemination and higher 1-year mortality among immunocompetent patients with chronic lung disease may reflect delayed recognition of extrapulmonary spread in a population where symptoms are often attributed to underlying pulmonary pathology. In addition, chronic inflammatory remodeling and impaired local immunity could hinder bacterial clearance once dissemination occurs. In contrast, immunocompromised patients are likely evaluated sooner and more aggressively [5, 7]. Dissemination may also represent a more aggressive or invasive phenotype among the chronic lung disease population, who otherwise commonly have slowly progressive, pulmonary nocardiosis. Microbiologic factors such as *N. farcinica* infection, variability in bacterial virulence factors, or suboptimal drug penetration may also contribute. These hypotheses warrant future mechanistic investigation.

Our study's limitations include its retrospective, single-center design, which risks selection bias. Not all patients had CNS imaging, which may have led to misclassification and underestimation of dissemination. However, sensitivity analyses excluding patients without CNS imaging showed similar results. The CCI overlaps with components of our risk-group definitions (eg, malignancy and chronic lung disease), introducing potential collinearity. We also had a relatively low number of patients with disseminated infection in the chronic lung disease group. Additionally, 38% of immunocompromised patients had chronic lung disease, but subgroup analyses were underpowered to assess differential effects. Incomplete follow-up for some patients may have led to underestimation of late complications or mortality. Variability in diagnostic evaluation, including inconsistent CNS imaging or delayed microbiologic testing, may have contributed to under-recognition of disseminated infection. Current guidelines recommend more aggressive therapy for patients with disseminated infection. While combination therapy has not been definitively shown to improve outcomes, these differences in therapy may have affected mortality rates. AST was only performed upon provider request and patients with AST likely had worrisome *Nocardia* species, presence of invasive disease, or involvement of anatomically challenging sites. Furthermore, while our cohort was relatively large and included diverse patient groups, some analyses were likely underpowered.

## CONCLUSIONS

Immunocompromised patients, immunocompetent individuals without chronic lung disease, and those infected with *N. farcinica* are at increased risk for disseminated nocardiosis. Dissemination was associated with increased 1-year mortality only among immunocompetent patients with chronic lung disease. These findings emphasize that host factors, rather than infection-specific variables, drive prognostic differences. Recognition of these patterns may guide early imaging decisions and individualized management strategies. Future studies should refine risk stratification tools and evaluate host–pathogen interactions to better inform long-term outcomes.

Immunocompetent patients with chronic lung disease may not require routine brain imaging in the absence of neurologic symptoms, though larger studies are needed before this can inform clinical recommendations. Future studies should re-evaluate risk stratification and host–pathogen interactions to better guide early imaging, therapeutic intensity, and long-term management strategies.

## Supplementary Material

ofag008_Supplementary_Data

## Supplementary Data

Supplementary materials are available at Open Forum Infectious Diseases online. Consisting of data provided by the authors to benefit the reader, the posted materials are not copyedited and are the sole responsibility of the authors, so questions or comments should be addressed to the corresponding author.
