# Supplementary material for: Risk Factors and Outcomes of Disseminated Nocardiosis Across Host Risk Groups
Source: Open Forum Infect Dis. 2026 Jan 12;13(1):ofag008. doi: 10.1093/ofid/ofag008 (PMC12817985; doi:10.1093/ofid/ofag008)
Supplement: ofag008_Supplementary_Data [file ofag008_supplementary_data.docx]

**Risk Factors and Outcomes of Disseminated Nocardiosis Across Host Risk Groups**

**Supplementary Materials**

Maria Vega Brizneda, MD^1^; Cyndee Miranda, MD^1^; Eric Cober, MD^1^; Anisha Misra, PhD^2^; Susan Harrington, PhD^2^; Zachary A. Yetmar, MD^1^

^1^Department of Infectious Disease, Cleveland Clinic, Cleveland, Ohio, USA

^2^Department of Pathology and Laboratory Medicine, Cleveland Clinic, Cleveland, Ohio, USA

Supplementary Table 1: Mutually exclusive sites of *Nocardia* infection

| Dissemination |  | Overall (N=232) |
| --- | --- | --- |
| **No** | Arthritis | 2 (1.1%) |
|  | Bacteremia | 2 (1.1%) |
|  | Cutaneous | 35 (18.6%) |
|  | Empyema | 1 (0.5%) |
|  | Epidural abscess | 1 (0.5%) |
|  | Intra-abdominal abscess | 1 (0.5%) |
|  | Keratitis | 1 (0.5%) |
|  | Lymphadenitis | 1 (0.5%) |
|  | Mediastinitis | 1 (0.5%) |
|  | Osteomyelitis | 1 (0.5%) |
|  | Perinephric abscess | 1 (0.5%) |
|  | Peritonitis | 1 (0.5%) |
|  | Pleuropulmonary | 9 (4.8%) |
|  | Pulmonary | 126 (67.0%) |
|  | Sinusitis | 1 (0.5%) |
|  | Skin + bursitis | 1 (0.5%) |
|  | Skin + osteomyelitis | 1 (0.5%) |
|  | Skin + pyomyositis | 1 (0.5%) |
|  | Submandibular abscess | 1 (0.5%) |
| **Yes** | CNS | 7 (15.9%) |
|  | CNS + epidural abscess + psoas abscess | 1 (2.3%) |
|  | CNS + skin + bacteremia + retinitis | 1 (2.3%) |
|  | Pleuropulmonary + bacteremia | 1 (2.3%) |
|  | Pleuropulmonary + CNS | 1 (2.3%) |
|  | Pleuropulmonary + CNS + bacteremia | 1 (2.3%) |
|  | Pleuropulmonary + CNS + bacteremia + endophthalmitis | 1 (2.3%) |
|  | Pleuropulmonary + CNS + osteomyelitis + endophthalmitis | 1 (2.3%) |
|  | Pleuropulmonary + skin + bacteremia | 1 (2.3%) |
|  | Pulmonary + bacteremia | 4 (9.1%) |
|  | Pulmonary + CNS | 12 (27.3%) |
|  | Pulmonary + CNS + pyomyositis | 1 (2.3%) |
|  | Pulmonary + CNS + skin | 7 (15.9%) |
|  | Pulmonary + epidural abscess | 1 (2.3%) |
|  | Pulmonary + liver abscess | 1 (2.3%) |
|  | Pulmonary + skin | 3 (6.8%) |

Abbreviation: CNS, central nervous system.

Supplementary Table 2: *Nocardia* species distribution by clinical syndrome

| ***Nocardia* species** | **Disseminated** | | **Non-disseminated** | | | **Total (N=232)** |
| --- | --- | --- | --- | --- | --- | --- |
|  | **CNS (N=33)** | **Non-CNS (N=11)** | **Cutaneous (N=38)** | **Pulmonary (N=136)** | **Other (N=14)** |  |
| *N. abscessus* | 4 (12.1%) | 1 (9.1%) | 1 (2.6%) | 19 (14.0%) | 3 (21.4%) | 28 (12.1%) |
| *N. amikacinotolerans* | 0 (0.0%) | 0 (0.0%) | 0 (0.0%) | 1 (0.7%) | 0 (0.0%) | 1 (0.4%) |
| *N. araoensis* | 0 (0.0%) | 0 (0.0%) | 0 (0.0%) | 1 (0.7%) | 0 (0.0%) | 1 (0.4%) |
| *N. asteroides* | 0 (0.0%) | 0 (0.0%) | 0 (0.0%) | 2 (1.5%) | 0 (0.0%) | 2 (0.9%) |
| *N. beijingensis* | 1 (3.0%) | 1 (9.1%) | 0 (0.0%) | 2 (1.5%) | 0 (0.0%) | 4 (1.7%) |
| *N. brasiliensis* | 0 (0.0%) | 0 (0.0%) | 22 (57.9%) | 1 (0.7%) | 1 (7.1%) | 24 (10.3%) |
| *N. concava* | 0 (0.0%) | 0 (0.0%) | 0 (0.0%) | 1 (0.7%) | 0 (0.0%) | 1 (0.4%) |
| *N. cyriacigeorgica* | 6 (18.2%) | 1 (9.1%) | 0 (0.0%) | 25 (18.4%) | 1 (7.1%) | 33 (14.2%) |
| *N. farcinica* | 12 (36.4%) | 3 (27.3%) | 3 (7.9%) | 19 (14.0%) | 3 (21.4%) | 40 (17.2%) |
| *N. inohanensis* | 1 (3.0%) | 0 (0.0%) | 0 (0.0%) | 0 (0.0%) | 0 (0.0%) | 1 (0.4%) |
| *N. niwae* | 1 (3.0%) | 0 (0.0%) | 0 (0.0%) | 0 (0.0%) | 0 (0.0%) | 1 (0.4%) |
| *N. nova* | 3 (9.1%) | 3 (27.3%) | 4 (10.5%) | 46 (33.8%) | 5 (35.7%) | 61 (26.3%) |
| *N. otitidiscaviarum* | 1 (3.0%) | 0 (0.0%) | 1 (2.6%) | 1 (0.7%) | 0 (0.0%) | 3 (1.3%) |
| *N. paucivorans* | 1 (3.0%) | 0 (0.0%) | 0 (0.0%) | 1 (0.7%) | 0 (0.0%) | 2 (0.9%) |
| *N. pseudobrasiliensis* | 1 (3.0%) | 1 (9.1%) | 0 (0.0%) | 0 (0.0%) | 0 (0.0%) | 2 (0.9%) |
| *N. puris* | 0 (0.0%) | 0 (0.0%) | 0 (0.0%) | 1 (0.7%) | 0 (0.0%) | 1 (0.4%) |
| *N. testacea* | 0 (0.0%) | 0 (0.0%) | 0 (0.0%) | 1 (0.7%) | 0 (0.0%) | 1 (0.4%) |
| *N. transvalensis* | 0 (0.0%) | 0 (0.0%) | 1 (2.6%) | 0 (0.0%) | 0 (0.0%) | 1 (0.4%) |
| *N. veterana* | 1 (3.0%) | 0 (0.0%) | 0 (0.0%) | 0 (0.0%) | 0 (0.0%) | 1 (0.4%) |
| *N. vinacea* | 0 (0.0%) | 0 (0.0%) | 1 (2.6%) | 0 (0.0%) | 0 (0.0%) | 1 (0.4%) |
| *N. vulneris* | 0 (0.0%) | 0 (0.0%) | 3 (7.9%) | 0 (0.0%) | 0 (0.0%) | 3 (1.3%) |
| *Nocardia* species | 1 (3.0%) | 1 (9.1%) | 2 (5.3%) | 15 (11.0%) | 1 (7.1%) | 20 (8.6%) |

Abbreviation: CNS, central nervous system.

*Nocardia* species are isolates who were unable to be identified to the species level.

Supplementary Table 3: *In vitro* antimicrobial susceptibility for 186 *Nocardia* isolates

| Antimicrobial | Number (% Susceptible) |
| --- | --- |
| **Amikacin** | 183 (98.4%) |
| **Amoxicillin-clavulanate** | 71 (38.2%) |
| **Ceftriaxone** | 121 (65.1%) |
| **Ciprofloxacin** | 27 (14.5%) |
| **Clarithromycin** | 82 (44.1%) |
| **Doxycycline** | 32 (17.2%) |
| **Imipenem** | 116 (62.4%) |
| **Linezolid** | 186 (100.0%) |
| **Minocycline** | 54 (29.0%) |
| **Moxifloxacin** | 71 (38.2%) |
| **Tobramycin** | 96 (51.6%) |
| **Trimethoprim-sulfamethoxazole** | 186 (100.0%) |

*Nocardia* spp. with an MIC in the intermediate or resistant range were considered non-susceptible.

Supplementary Table 4: Initial antibiotic therapy for nocardiosis

|  | Non-disseminated  (N=188) | Disseminated  (N=44) | Total  (N=232) | P-value |
| --- | --- | --- | --- | --- |
| Amikacin | 5 (2.7%) | 7 (15.9%) | 12 (5.2%) | 0.002 |
| Amoxicillin-clavulanate | 8 (4.3%) | 0 (0.0%) | 8 (3.4%) | 0.358 |
| Ceftriaxone | 12 (6.4%) | 6 (13.6%) | 18 (7.8%) | 0.119 |
| Ciprofloxacin | 4 (2.1%) | 1 (2.3%) | 5 (2.2%) | 1.000 |
| Clarithromycin | 2 (1.1%) | 0 (0.0%) | 2 (0.9%) | 1.000 |
| Doxycycline | 3 (1.6%) | 0 (0.0%) | 3 (1.3%) | 1.000 |
| Imipenem | 23 (12.2%) | 19 (43.2%) | 42 (18.1%) | < 0.001 |
| Linezolid | 13 (6.9%) | 14 (31.8%) | 27 (11.6%) | < 0.001 |
| Minocycline | 11 (5.9%) | 2 (4.5%) | 13 (5.6%) | 1.000 |
| Moxifloxacin | 2 (1.1%) | 0 (0.0%) | 2 (0.9%) | 1.000 |
| Trimethoprim-sulfamethoxazole | 148 (78.7%) | 31 (70.5%) | 179 (77.2%) | 0.238 |
| Meropenem | 28 (14.9%) | 14 (31.8%) | 42 (18.1%) | 0.015 |
| Azithromycin | 2 (1.1%) | 0 (0.0%) | 2 (0.9%) | 1.000 |
| Tigecycline | 1 (0.5%) | 0 (0.0%) | 1 (0.4%) | 1.000 |
| Number of initial agents | 1.0 (1.0, 2.0) | 2.0 (2.0, 2.0) | 1.0 (1.0, 2.0) | < 0.001 |
| One antibiotic | 115 (61.2%) | 5 (11.4%) | 120 (51.7%) | < 0.001 |
| Two antibiotics | 67 (35.6%) | 29 (65.9%) | 96 (41.4%) | < 0.001 |
| Three antibiotics | 5 (2.7%) | 10 (22.7%) | 15 (6.5%) | < 0.001 |
| Combination therapy | 72 (38.3%) | 39 (88.6%) | 111 (47.8%) | < 0.001 |
| Number of active initial agents | 1.0 (1.0, 2.0) | 2.0 (2.0, 2.0) | 1.0 (1.0, 2.0) | < 0.001 |
| Length of therapy | 190.0 (99.0, 288.0) | 364.0 (252.0, 391.0) | 204.5 (112.0, 339.0) | < 0.001 |
| Procedural intervention for Nocardia | 34 (18.1%) | 25 (56.8%) | 59 (25.4%) | < 0.001 |
| Secondary prophylaxis | 28 (19.2%) | 13 (41.9%) | 41 (23.2%) | 0.010 |

Data are N (%) or median (interquartile range) for categorical or continuous variables, respectively.

Supplementary Table 5: Multivariable logistic regression model of associations with disseminated infection, excluding patients without brain imaging

|  | **Odds ratio** | **95% CI** | **P-value** |
| --- | --- | --- | --- |
| **Immunocompetent without lung disease^1^** | 6.83 | 2.06-27.42 | 0.003 |
| **Immunocompromised^1^** | 6.56 | 2.14-25.07 | 0.002 |
| ***Nocardia farcinica*** | 2.72 | 1.12-6.69 | 0.027 |
| **Charlson comorbidity index (per 1 point increase)** | 1.02 | 0.83-1.25 | 0.812 |
| **Primary trimethoprim-sulfamethoxazole prophylaxis** | 0.47 | 0.12-1.59 | 0.252 |

Abbreviations: CI, confidence interval.

^1^Compared to the chronic lung disease group. This analysis was repeated with the initial 3-level risk category variable recategorized as “Immunocompetent with chronic lung disease”. This group showed significantly reduced odds of dissemination (odds ratio 0.15, 95% confidence interval 0.03-0.42; *p*=0.001).

Supplementary Table 6: Multivariable logistic regression model of associations with disseminated infection, excluding immunocompetent patients with cutaneous nocardiosis

|  | **Odds ratio** | **95% CI** | **P-value** |
| --- | --- | --- | --- |
| **Immunocompetent without lung disease^1^** | 10.22 | 3.00-41.53 | < 0.001 |
| **Immunocompromised^1^** | 7.24 | 2.46-26.75 | < 0.001 |
| ***Nocardia farcinica*** | 3.19 | 1.33-7.66 | 0.009 |
| **Charlson comorbidity index (per 1 point increase)** | 1.19 | 0.98-1.45 | 0.082 |
| **Primary trimethoprim-sulfamethoxazole prophylaxis** | 0.64 | 0.16-2.12 | 0.487 |

Abbreviations: CI, confidence interval.

^1^Compared to the chronic lung disease group. This analysis was repeated with the initial 3-level risk category variable recategorized as “Immunocompetent with chronic lung disease”. This group showed significantly reduced odds of dissemination (odds ratio 0.12, 95% confidence interval 0.03-0.34; *p*<0.001).

Supplementary Table 7: Multivariable Cox regression model of associations with one-year mortality excluding patients without brain imaging

|  | **Hazard ratio** | **95% CI** | **P-value** |
| --- | --- | --- | --- |
| **Immunocompetent without lung disease^1^** | 1.76 | 0.38-8.11 | 0.468 |
| **Immunocompromised^1^** | 3.33 | 0.94-11.82 | 0.063 |
| **Dissemination** | 1.62 | 0.72-3.65 | 0.242 |
| **Charlson comorbidity index (per 1 point increase)** | 1.24 | 1.05-1.45 | 0.010 |
| ***Nocardia farcinica*** | 1.09 | 0.42-2.83 | 0.861 |

Abbreviations: CI, confidence interval.

^1^Compared to the chronic lung disease group.

Supplementary Table 8: Multivariable Cox regression model of associations with one-year mortality excluding immunocompetent patients with cutaneous nocardiosis

|  | **Hazard ratio** | **95% CI** | **P-value** |
| --- | --- | --- | --- |
| **Immunocompetent without lung disease^1^** | 2.14 | 0.63-7.26 | 0.221 |
| **Immunocompromised^1^** | 3.57 | 1.42-8.98 | 0.007 |
| **Dissemination** | 1.20 | 0.57-2.53 | 0.622 |
| **Charlson comorbidity index (per 1 point increase)** | 1.26 | 1.09-1.46 | 0.002 |
| ***Nocardia farcinica*** | 1.32 | 0.58-3.03 | 0.508 |

Abbreviations: CI, confidence interval.

^1^Compared to the chronic lung disease group.
